# Computational design of fully overlapping coding schemes for protein pairs and triplets

**DOI:** 10.1038/s41598-017-16221-8

**Published:** 2017-11-20

**Authors:** Vaitea Opuu, Martin Silvert, Thomas Simonson

**Affiliations:** Laboratoire de Biochimie (CNRS UMR7654), Ecole Polytechnique, Palaiseau, France

## Abstract

Gene pairs that overlap in their coding regions are rare except in viruses. They may occur transiently in gene creation and are of biotechnological interest. We have examined the possibility to encode an arbitrary pair of protein domains as a dual gene, with the shorter coding sequence completely embedded in the longer one. For 500 × 500 domain pairs (X, Y), we computationally designed homologous pairs (X′, Y′) coded this way, using an algorithm that provably maximizes the sequence similarity between (X′, Y′) and (X, Y). Three schemes were considered, with X′ and Y′ coded on the same or complementary strands. For 16% of the pairs, an overlapping coding exists where the level of homology of X′, Y′ to the natural proteins represents an E-value of 10^−10^ or better. Thus, for an arbitrary domain pair, it is surprisingly easy to design homologous sequences that can be encoded as a fully-overlapping gene pair. The algorithm is general and was used to design 200 triple genes, with three proteins encoded by the same DNA segment. The ease of design suggests overlapping genes may have occurred frequently in evolution and could be readily used to compress or constrain artificial genomes.

## Introduction

Overlapping gene pairs are found in many viruses and organisms^1–5^. In higher organisms, the overlapping regions usually involve introns, 5′- or 3′-untranslated regions, or very short protein coding segments. Few examples of long, overlapping, protein coding segments have been described in bacteria, yeast, or mitochondria^1,2,6^. Thus, a survey of prokaryotic genomes found just six pairs of genes with overlaps of 30 amino acids or more^1^. Only in viruses is it common to find protein coding segments that overlap extensively. Indeed, coding two proteins on one stretch of DNA severely limits the coding possibilities. If the proteins are coded on the same strand in different reading frames, each nucleotide is part of two overlapping codons; if they are coded on opposite strands, the codons used for each protein must base pair. These constraints affect the rate of genetic drift, limit the ability to become optimally adapted, and presumably explain the counterselection of such sequences.

Nevertheless, the possibility of forming overlapping pairs of protein coding regions has biological importance for several reasons. In viruses, it is presumably a response to selective pressure for a reduced genome size, compatible with a fixed capsid volume. It also leads to constraints on protein regulation and expression. Indeed, two proteins coded on opposite strands would be hard to transcribe at the same time because of collisions between the two polymerases, and the transcript of one would be able to base pair with the transcript of the other to form double-stranded RNA. It may be that the few observed bacterial, yeast, and mitochondrial instances have survived because of such specific regulatory properties^1,2,4,6^.

It has also been proposed that new genes could appear through a scenario where an alternate reading frame within an existing gene acquires the ability to be transcribed and translated into a polypeptide, which could eventually become functional^1,7,8^. Later, gene duplication would create a second copy of the overlapping pair, and within each copy, one of the genes would become defunct. In particular, viruses are thought to use alternate reading frames, either on the antisense or the sense strand, to generate new genes^9^, a mechanism known as “overprinting”^10^. A special case of transient dual genes could be when horizontal transfer of an overlapping gene pair occurs from a virus to a cellular organism. The hypothesis of spontaneous antisense gene appearance has found support in the structure of the genetic code^11^. It has been pointed out that with both the usual and the mitochondrial genetic codes, the nucleotide triplets that encode polar (respectively, nonpolar) amino acids are complementary to antisense triplets that encode nonpolar (polar) amino acids. In addition, the secondary structure preferences of the sense/antisense amino acids are similar. This complementarity could facilitate the folding and gain of function of a hypothetical antisense polypeptide. A special case of this hypothesis is that peptide ligands might have arisen for some proteins from the antisense strand complementary to their coding region^11,12^.

In addition to their biological importance, overlapping genes could be of significant interest in biotechnology, to compress or constrain artificial genomes. In particular, a synthetic organism where several genes overlap would have fewer available neutral mutations, since each mutation in the overlapping region would affect more than one protein. Thus, the organism would undergo reduced genetic drift and remain closer to its original, designed genotype. An example of a designed overlapping gene was produced recently, where each DNA strand coded for a simplified but functional aminoacyl-tRNA synthetase “Urzyme”^13–16^, with the two enzymes being homologous to the two modern synthetase classes.

**Figure 1:**
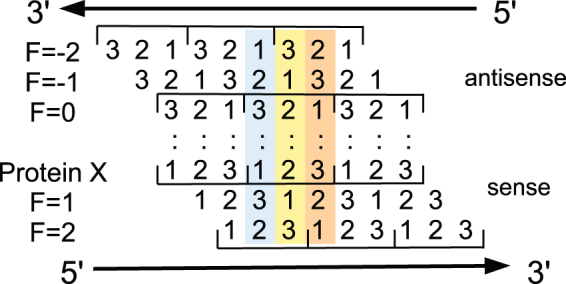
The five possible readings frames *F* for protein Y, relative to the X frame.

We have developed a general method to design overlapping genes, with two main objectives: (1) to expand our ability to engineer artificial genes and genomes and (2) to help evaluate the importance of overlapping genes in evolution. Indeed, to evaluate their role in evolution, we should have a precise idea of the difficulty to create them. The rarity of contemporary examples and the stringent overlap constraints suggest that it is very difficult. To quantify in a general way the difficulty to create overlapping genes, we have examined the possibility of encoding an arbitrary pair of protein domains in a single DNA segment, as a fully-overlapping dual gene. We considered three possible reading frames for the second protein, relative to the first: two on the antisense strand and one on the sense strand. Indeed, sense and antisense overlap schemes have different implications for the biological hypotheses above and different capabilities to code a variety of amino acid types^11,17^. One sense:antisense overlap scheme placed the third, wobble base in each codon opposite the central nucleotide of another codon (Fig. 1). Given the structure of the genetic code, this is expected to be the most favorable scheme, as confirmed below. Another scheme placed the central bases of the sense and antisense codons opposite each other (*F* = 0 scheme, Fig. 1). This is less favorable, since each central base constrains the other. The two sense:sense overlap schemes are similar and we chose one of them.

We considered 500 protein domains from the Pfam database^18^, 70–100 amino acids long, and all 125,250 corresponding domain pairs (X, Y). 44 of the proteins (9%) were viral proteins. For each (X, Y) pair, we searched for two homologs (X′, Y′) that can be encoded in a fully overlapping manner. We required that the coding sequence of the smaller domain be completely embedded within that of the larger one. For each pair, we explored three possible overlap schemes, where X′ and Y′ are encoded either on the same DNA strand in two different reading frames or on opposite strands. The search used a dynamic programming scheme^19^, presented here, that provably maximizes a total X/X′ and Y/Y′ similarity score. The method’s cost is proportional to the length of the shorter protein, allowing a large-scale study. Considering all three reading frames, we found that over 16% of the (X, Y) pairs have homologous pairs (X′, Y′) that can be encoded in a fully overlapping manner, with a level of X/X′ and Y/Y′ homology corresponding to Blast E-values of 10^−10^ or less and a match length of at least 85% of the total sequence length. The success rate was 14% for pairs of non-viral proteins and 52% for pairs of viral proteins. None of the 502 viral pairs were natural pairs occurring within the same virus. Thus, it appears that many pairs of protein domains have close homologs that can be encoded by a fully-overlapping dual gene and the tendency is especially high for viral proteins. This is in striking contrast to naive expectation. Differences between the proteins that do or do not allow overlapping schemes are analyzed and discussed.

Given the level of success and the generality of our algorithm, we also designed 200 *triple* genes, with three proteins encoded by the same DNA segment using both strands and three different reading frames. All the designed proteins had E-values better than 10^−20^ against their natural homologs. The designs included 62 triplets of bacterial proteins and 30 triplets of viral proteins. Such triple overlaps have been found in viruses^9,20,21^, but the corresponding protein sequences may be unstructured, in contrast to our designed sequences. It has also been proposed that triple overlaps may have existed in ancestral ribosome sequences^22^. The ease of design revealed here of both double and triple genes is consistent with the overprinting mechanism for gene creation and suggests that overlapping genes could have occurred frequently in evolution. To facilitate their use in artificial genomes, our design code is provided online (see Github).

## Methods

### Designing overlapping homologs: goal of the algorithm

We start from a pair of protein domains, whose amino acid sequences we denote X and Y. The goal is to determine homologous amino acid sequences X′, Y′ such that the coding sequence of the smaller protein is entirely embedded within that of the larger protein, being coded either on the same DNA strand in a different reading frame or on the opposite strand. The five choices of reading frames for Y are shown in Fig. 1. We choose a reading frame *F* = −2, −1, 0, 1, or 2 for Y, and a starting point for the smaller of the two proteins, such that its coding sequence will be entirely embedded within that of the larger protein. Applying a sequence optimization algorithm described below, we obtain the protein sequences (X′, Y′), which are as similar as possible to X, Y and whose coding sequences completely overlap. The similarity is measured by a Blosum similarity score, summed over the length of both proteins:

1 $$S(X^{\prime} ,Y^{\prime} )=\sum _{i}\,{p}_{i}B({X}_{i},{X}_{i}^{^{\prime} })+\sum _{j}\,{q}_{j}B({Y}_{j},{Y}_{j}^{^{\prime} }),$$

where *B* is a Blosum scoring matrix, *X*_*i*_ (resp., *Y*_*i*_, ${X}_{i}^{^{\prime} }$, ${Y}_{i}^{^{\prime} }$) is the *i* ^*th*^ amino acid of X (resp., Y, X′, Y′), and *p*_*i*_, *q*_*j*_ are position-dependent weights that reflect sequence conservation among natural homologs of X and Y. They are chosen so that positions in X, Y that are highly conserved (respectively, variable) have high (low) weights (see below).

### Designing overlapping homologs: an exact algorithm

We now describe the optimization scheme, starting with the case *F* = 0. In this reading frame, Y′ is coded on the antisense strand in register with X′: each codon of Y′ overlaps with a single X′ codon on the opposite strand. To maximize the similarity score (Eq. (1)), we simply choose the optimal nucleotides for each pair of base-paired codons, by comparing the 64 possible choices and picking the one that gives the largest contribution to *S*(*X*′, *Y*′).

**Figure 2:**
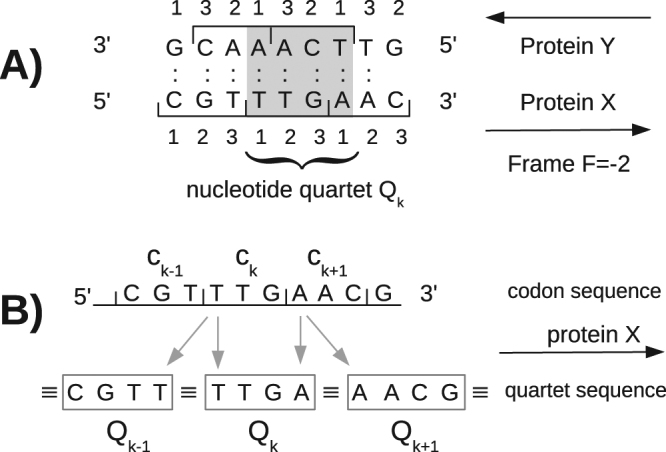
(**A**) A nucleotide quartet. (**B**) DNA sequence represented as a series of codons or linked quartets. The first nucleotide of each X codon appears twice in the sequence of quartets. Only the X strand is shown, for simplicity.

In all the other reading frames, each codon of X′ and Y′ overlaps with two other codons and another approach is needed. We consider the *F* = −2 case first. The DNA sequence to be optimized is the region of X′, Y′ overlap. We number the X′ codons in this region in the 5′ → 3′ direction (the direction of the polypeptide sequence). For each X′ codon *c*_*X*_(*k*), we denote *c*_*Y*_(*k*) the Y′ codon on the opposite strand that has two overlapping nucleotides. The two codons *c*_*X*_(*k*) and *c*_*Y*_(*k*) define a quartet of nucleotides on the X′ strand, which we denote *Q*_*k*_, shown in Fig. 2A. The following quartet *Q*_*k*+1_ shares its first nucleotide with *Q*_*k*_: *Q*_*k*+1_(1) = *Q*_*k*_(4), so that the sequences of any two consecutive quartets are linked^17^. The key step is to express the DNA sequence, not as a series of codons but as a series of quartets, whose sequences will be varied subject to the linkage constraint. This representation of the DNA sequence is shown in Fig. 2B.

**Figure 3:**
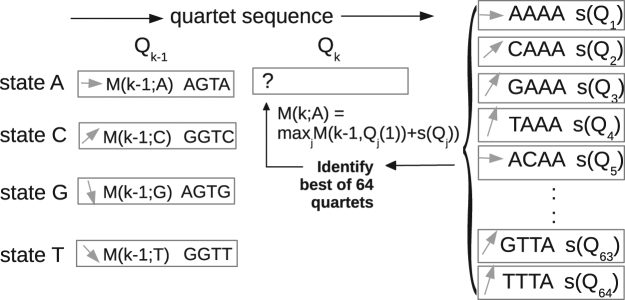
Dynamic programming algorithm for sequence design. Columns *k* − 1 and *k* are consecutive positions in the sequence of quartets. The boxes in these columns correspond to states ${\mathscr{S}}$ = A, C, G, or T. The *k*−1 states are each annotated with an optimal quartet *Q*, a pointer indicating the previous state, and the score $M(k-\mathrm{1,}\,{\mathscr{S}})$ of the best sequence that terminates in that state. To annotate the A state in column *k*, we consider the 64 quartets on the right and select the one that leads to the maximal score, $M(k,{\mathscr{S}}=A)$. Q_j_(1) is the first nucleotide of quartet j. The pointer in each box (grey arrow) indicates by its slope which is the previous state; for example the pointer in the state G box on the left comes from state A at the very top of the *k* − 2 column; the pointer in the state T box comes from the G state one line above in the *k* − 2 column.

With this representation, the problem of maximizing *S*(*X*′, *Y*′) is closely analogous to sequence alignment^19^ and can be solved by a similar recursive method, schematized in Fig. 3. Let *N* be the number of X′ codons, which is also the number of quartets. For any position *k* in the sequence, there are 4^4^ = 256 possible quartets *Q*, which can be subdivided into four groups of 64, depending on their last nucleotide: *Q*(4) = A, C, G, or T. This nucleotide defines the “state” of the quartet: ${\mathscr{S}}(Q)\equiv Q\mathrm{(4)}$. The first step of our algorithm consists in filling a 4-by-*N* table of optimal scores, where each entry corresponds to a position in the sequence of quartets and a quartet state. We fill the table from left to right, one column at a time. To fill in column *k*, we assume column *k* − 1 contains, for each state ${\mathscr{S}}$, the score of the optimal sequence that terminates in this state, say $M(k-\mathrm{1;}\,{\mathscr{S}})$. For column *k*, for any quartet *Q*, an optimal sequence terminating with *Q* is necessarily obtained by adding *Q* to a sequence whose *k* − 1 quartet has the state ${\mathscr{S}}$ = *Q*(1). Let *s*(*Q*) be the contribution to *S*(*X*′, *Y*′) of the two codons contained within a quartet *Q* (and its complementary strand). We have the recursion:

2 $$M(k;{\mathscr{S}})=\mathop{{\rm{\max }}}\limits_{{Q}_{j}\in {\mathscr{S}}}\,\{M(k-\mathrm{1;}\,{\mathscr{S}}^{\prime} \equiv {Q}_{j}\mathrm{(1)})+s({Q}_{j})\}$$

The maximum is taken over the 64 quartets *Q*_*j*_ that have ${\mathscr{S}}$ as their last nucleotide (${Q}_{j}\in {\mathscr{S}}$). Their optimal character is tested by adding *s*(*Q*_*j*_) to the score $M(k-1;{\mathscr{S}}^{\prime} \equiv {Q}_{j}\mathrm{(1))}$ from the previous column, where ${\mathscr{S}}^{\prime}$ is the state *Q*_*j*_ is linked to.

As we fill in entry $M(k;{\mathscr{S}})$ in the table, we also tabulate the “optimal” quartet, called *Q*_*k*_, that led to its score. Initialization of the leftmost column is done by adding a column “0” to the left of the table, filling it with zeroes, and applying (2) to column *k* = 1. Once the table is filled, we perform a traceback operation, analogous to sequence alignment. It consists in concatenating the optimal quartets backwards, with the first nucleotide *Q*_*k*_(1) of the each optimal quartet serving as a pointer to the state ${\mathscr{S}}$ we should move to in the previous column. The cost of the whole procedure is proportional to the length *N* of the overlapping sequences. For the reading frames other than *F* = −2, the same method applies; only the positions of the X′ and Y′ codons within each quartet *Q* change, changing slightly the mechanics for calculating the score *S*(*Q*). This method leads to the sequences X′, Y′ that globally maximize the score *S*(*X*′, *Y*′).

The method also applies, with straightforward adjustments, to cases where each nucleotide quartet contains three or more overlapping codons; for example, a reference X codon, a second Y codon on the opposite strand (*F* = −2 frame) and a third Z codon on the same strand (*F* = 1 frame). Only the quartet scoring mechanics (Eq. (1)) change slightly, with each codon in the quartet contributing a term to the score *S*(*X*′, *Y*′, *Z*′). The method can thus be used to design three, four, or even six overlapping genes. To design five or six overlapping genes (using all six reading frames at once), the coding sequence should be represented as a series of nucleotide quintets, instead of quartets. It is also straightforward to extend the algorithm to situations where one uses more than four nucleotide types, or codons that are four nucleotides long, instead of three^23^, which could be of interest in synthetic biology, or still longer codons, which could be of interest for other types of information storage. Below, we present the successful design of 200 triple genes as an illustration.

### Test data and calculations

The algorithm was tested on 500 Pfam protein domains, 70–100 amino acids long, from 500 Pfam families. Within each family, the first protein was chosen. Each domain was assigned a conservation pattern based on the Pfam seed alignment of its family. Specifically, we assigned to each position *i* of X a weight *p*_*i*_, inversely proportional to the exponential of the sequence entropy *S*_*i*_ of the corresponding column^19^. The entropy was computed not from the amino acid types, but from a simplified classification into six groups: {LVIMC}, {FYW}, {G}, {ASTP}, {EDNQ}, and {KRH}. Thus, ${e}^{{S}_{i}}$ takes values between 1 and 6.

The search algorithm was applied to all 500 × 500 domain pairs, in three possible reading frames. For the frames *F* = −2 and *F* = 0, we did two calculations per pair, with the beginning of the shorter protein X′ aligned with either end of Y′. For the frame *F* = 1, we also did two calculations per pair; the beginning of the two proteins were aligned, and either Y or X was in the *F* = 1 frame (with the other protein in the *F* = 0 frame). This led to a total of 751,500 optimization runs. The nucleotide sequences do not include a STOP codon; we assume one can be added (to the shorter protein) by manually changing a few nucleotides.

To evaluate the similarity between the homologs X′, Y′ and the target proteins X, Y, we did a Blast search of the Swissprot database using the homolog sequence X′ or Y′ as the query, and retrieved the best E-value corresponding to the target protein family. This was not always the target protein X or Y, but sometimes a natural homolog from the same Pfam family. The corresponding E-values for X′ and Y′ were compared, and the largest one, denoted *E*_*p*_ (X′, Y′), was taken as the homology metric. For the longer protein, say Y, the non-overlapping part of the protein sequence was not included in the Blast query; only the overlapping sequence was employed. We only considered a Blast result if the match length was at least 85% of the length of the overlapping sequence region; all shorter Blast matches were discarded.

To evaluate the similarity further, we submitted each homolog to the Superfamily library of Hidden Markov Models^24^, which each correspond to one family in the SCOP classification of protein domains^25^, to determine if the submitted sequence belongs to the correct SCOP family. Only the shorter protein of the pair was tested. Of the 500 natural Pfam domains, 183 had a SCOP match according to Superfamily; the Superfamily tests were limited to their predicted homologs.

## Results

For all 125,250 pairs of Pfam proteins (X, Y) and all three reading frames considered here, we used our search algorithm to find homologous sequences (X′, Y′) that are coded by two fully overlapping DNA sequences, with the shorter protein’s coding sequence completely embedded in the longer one’s. The similarity of the computed homologs to natural sequences from the same family as X or Y was characterized by their Blast E-values versus the Swissprot sequence collection. For each pair and reading frame, we made two predictions, corresponding to two different positions of the X′ 5′-terminus relative to Y′, for a total of 751,500 predictions. Blast hits were only counted if the match lengths represented at least 85% of the total sequence length.

**Figure 4:**
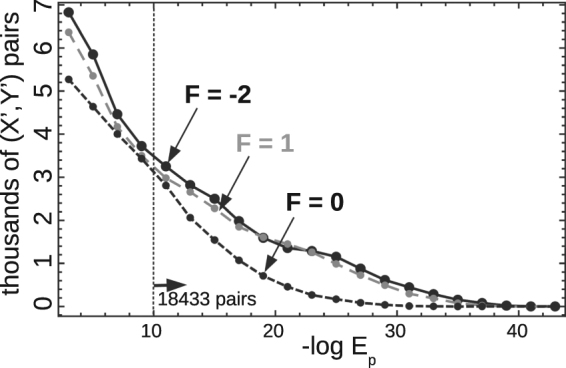
E-value histograms for the three reading frames. For each pair of homologs, the E-value corresponds to *E*_*p*_ (X′, Y′), the largest of the X′ and Y′ values. Of the 125,250 pairs, only those with E-values better than 10^−2^ are shown. A dashed vertical line indicates the −log *E*_*p*_ = 10 threshold; the number of pairs with E-values above this threshold is indicated.

The fractions of pairs with low E-values are reported in Table 1. Histograms of *E*_*p*_ values are shown in Fig. 4. Summing over all three reading frames, we obtained an *E*_*p*_(*X*′, *Y*′) value of 10^−10^ or better for 20,502 pairs, representing 16.3% of all pairs. For 12,162 pairs (9.7%) the *E*_*p*_(*X*′, *Y*′) value was 10^−15^ or better. For pairs of non-viral proteins, the success rate (*E*_*p*_(*X*′, *Y*′) value of 10^−10^ or better) was 14.1%. For pairs of viral proteins, it was especially high: 51.6%. None of the viral pairs were naturally-existing pairs. For viral/non-viral pairs, it was 26%. The success rate was best in the *F* = −2 frame and poorest in the *F* = 0 frame (7.4% of pairs with −log *E*_*p*_ > 10), which both correspond to sense/antisense overlap. This was expected, given the structure of the genetic code and the relative importance of the central and wobble codon bases (see below). For the *F* = 1 sense:sense overlap scheme, the success rate was intermediate: 13.5% of pairs had −log *E*_*p*_ > 10.

**Table 1:** Number of designed pairs with low Blast E-values.

−log(*E*_*p*_) range	pair type^a^	total number of pairs	Percentage of successful pairs overlap scheme			
			*F* = 0	*F* = 1	*F* = −2	All^b^
>10	v-v	990	33.2	48.1	50.7	51.6
>10	v-o	20520	8.4	22.4	24.3	26.0
>10	o-o	104196	6.9	11.3	12.4	14.1
>10	all	125250	7.4	13.5	14.7	16.3
>15	v-v	990	22.8	41.3	43.4	51.6
>15	v-o	20520	3.6	15.6	15.6	17.8
>15	o-o	104196	2.4	6.2	6.9	7.7
>15	all	125250	2.8	8.0	8.8	9.7

^a^Pairs types are viral-viral (v-v), viral-other (v-o), and other-other (o-o; i.e., two non-viral proteins). ^b^Including all 3 schemes.

Similar results were obtained from Superfamily searches. Of the 500 Pfam domains considered here, 183 were recognized by Superfamily as similar to a SCOP family. We considered the protein pairs (X, Y) where the shorter of the two, say X, is among these 183 domains, and where the predicted homolog pair (X′, Y′) has a low *E*_*p*_ value. We submitted each homolog X′ to Superfamily. Notice that we only considered the shorter protein, since the longer protein Y has non-overlapping amino acids that make it easier to recognize. Thus, X′ represents a more stringent test. For all three reading frames, over 83% of the homologs X′ were correctly recognized by Superfamily, further supporting their similarity to natural sequences.

**Figure 5:**
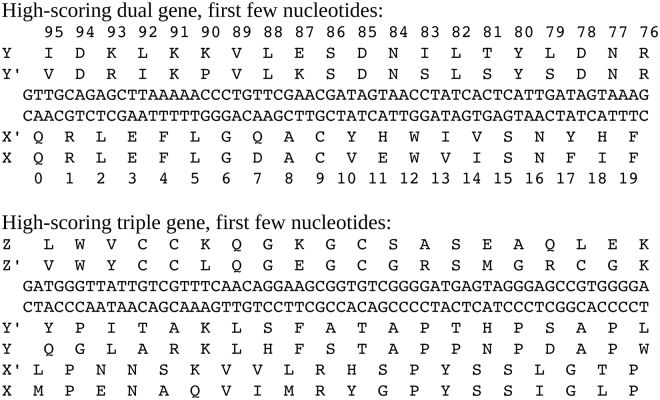
The first nucleotides of two genes. Above: a dual gene (*F* = −2) of bacterial proteins X = PF00636.25-RNC-UREPA/43-140 and Y = PF00636.25-RNC-UREPA/43-140. Below: a triple gene where X is the bovine protein PF15092.5-CJ053-BOVIN/1-89, Y is the viral protein PF04823.11-VP22-HHV11/170-256, and Z is the bovine tick protein PF10468.8-TCI1-RHIBU/10-96.

24 predictions corresponded to *E*_*p*_ values better than 10^−38^. Three of the pairs were (X, X) “self” pairs involving three bacterial proteins (Pfam families PF04379, PF20806, PF14173). Three were (X, Y) pairs involving bacterial proteins. The Pfam codes and −log *E*_*p*_ values for these pairs are: PF00635-PF04297 (40, 39.5); PF04297-PF05269 (38.7, 39.7); PF00166-PF14173 (38.0, 38.4). The Blosum62 similarity scores for these three pairs were in the range 314–346, for lengths of 98–100 amino acids. The beginning of the sequences for the first pair are shown in Fig. 5. Two other pairs, each involving one viral and one bacterial protein, gave −log *E*_*p*_ values above 40.

**Figure 6:**
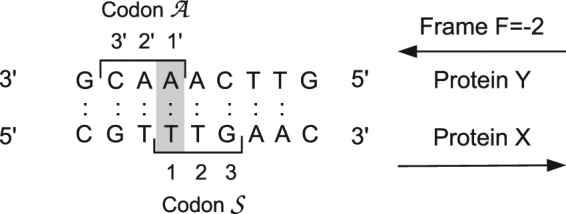
Overlapping sense/antisense codons in the *F* = −2 reading frame.

The *F* = −2 sense:antisense overlap scheme was the most favorable above, although *F* = 1 and *F* = 0 also gave many hits. In prokaryotic genomes, a slight preference for *F* = −2 was noted in surviving overlapping genes. Indeed, the coding properties of this scheme are especially favorable, and we analyze them briefly here. In this frame, each X′ codon has its middle nucleotide in register with a Y′ wobble nucleotide, and vice versa. Since the wobble nucleotide has little effect on the coded amino acid, this allows the middle nucleotide of each X′ and Y′ codon to be chosen almost freely. Figure 6 shows two sense/antisense codons that share their first bases. We denote them ${\mathscr{S}}$ and ${\mathscr{A}}$. We denote the sense nucleotides 1, 2, 3 and the antisense nucleotides 1′, 2′, 3′. The possible amino acid choices for ${\mathscr{S}}$ and ${\mathscr{A}}$ are listed in Table 2. If we classify the 20 amino acid types into four classes: Π = (large, polar), Φ = (large, nonpolar), *π* = (small, polar), *φ* = (small, nonpolar), with four choices of class on each strand, there are 16 possible ${\mathscr{S}}/{\mathscr{A}}$ combinations. Despite the base-pairing constraint, 15 of them can be encoded freely in this overlap scheme. Only the combination (*π*, *π*) cannot be encoded unambiguously. In contrast, the *F* = 0 frame (central bases opposite) can only encode 26 of 20 × 20 possible pairs of amino acid types.

**Table 2:** Sense/antisense amino acid (aa) types for overlapping codons, *F* = −2 reading frame.

**Amino acid classes produced by sense:antisense nucleotides**				
	nucleotide 2 or 2′			
	U	C	A	G
nt 1 = U	L, F ∈ Φ	S ∈ *π*	Y ∈ Φ, stop	C, W, stop
nt 1′ = A	I, M ∈ Φ	T ∈ *φ*	N, K ∈ Π	S, R
nt 1 = C	L ∈ Φ	P ∈ *φ*	H, Q ∈ Π	R ∈ Π
nt 1′ = G	V ∈ *φ*	A ∈ *φ*	D, E ∈ Π	G ∈ *π*
**Sense:antisense nucleotides needed to produce pairs of aa classes**				
**sense class**	**antisense (1′2′3′) aa class**			
	**Φ**	***φ***	**Π**	***π***
L, NP = Φ	U_1_U_2_:A_1′_U_2′_	U_1_U_2_:A_1′_C_2′_	U_1_U_2_:A_1′_A_2′_	A_1_U_2_:U_1′_C_2′_
S, NP = *φ*		C_1_C_2_:G_1′_U_2′_	G_1_U_2_:C_1′_A_2′_	G_1_C_2_:G_1′_G_2′_
L, P = Π			G_1_A_2_:C_1′_A_2′_	C_1_A_2_;G_1′_G_2′_
S, P = *π*			ambiguous → A_1_G_2_:U_1′_C_2′_	

The relation between the sense/antisense overlapping codons and the nucleotide numbers are defined in Fig. 6.

**Figure 7:**
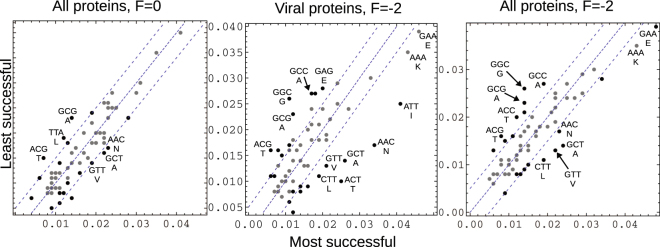
Codon frequencies in the “successful” and “unsuccessful” Pfam protein sets in two different overlap schemes. For the *F* = −2 scheme, the most successful among viral proteins (middle panel) or all proteins (right panel) are compared to the least successful among the whole Pfam set. Each dot in each panel represents a codon. Black dots correspond to the lowest P-values (P < 0.2) for the successful/unsuccessful differences. Dashed lines correspond to the main diagonal and diagonals offset by ±0.005. Selected codons are labelled with the corresponding nucleotides and amino acid.

To identify sequence properties that favor gene overlap, we characterized the 50 Pfam proteins that were most “successful,” participating most frequently in high scoring dual genes in the *F* = −2 frame (ones where the designed homologs had *E*_*p*_ < 10^−15^), as well as the 20 most successful viral proteins. We compared them to 142 “unsuccessful” Pfam proteins that gave no hits (all *E*_*p*_ values > 10^−2^). We repeated the analysis for the *F* = 0 and *F* = 1 frames. The *F* = −2 and *F* = 1 “successful” sets shared 43 proteins out of 50, so we only present the *F* = −2 and *F* = 0 sets. Tables 3 and 4 and Fig. 7 summarize the results. The “successful” proteins are slightly longer on average and the corresponding Pfam families are slightly less diverse, especially for the viral set (*F* = −2 scheme), with a mean entropy of 1.4, vs. 2.5 for the unsuccessful set (Table 3). The mean codon degeneracy and GC contents are very similar in all the successful and unsuccessful sets. There are a few differences in amino acid and 2-mer composition in the *F* = −2 scheme (Table 4), with F, W, Y, G depleted in both “successful” sets (viral and overall) and N enriched (P-values of 0.01–0.17), while the 2-mers DE, SN, SE are enriched and FA, FG, YR, YV depleted.

**Table 3:** Mean sequence properties of successful and unsuccessful Pfam protein sets.

Sequence property	*F* = −2 sets				*F* = 0 sets		
	^a^viral proteins	^b^most successful	^b^least successful	P-value^*c*^	^b^most successful	^b^least successful	P-value^*c*^
#pairs^*d*^	155.1	159.6	0		65.9	0	
length	85.8	89.2	83.8	9.10^−5^	87.7	83.6	3.10^−2^
entropy^*e*^	1.4	2.0	2.5	3.10^−4^	2.4	2.6	8.10^−2^
degeneracy^*f*^	352	350	350	0.9	349	352	0.4
GC%	45.5	46.5	49.2	0.2	48.6	48.9	0.9

^a^The 20 most successful out of 44. ^b^Out of all 500 Pfam proteins. ^c^Statistical significance of the successful/unsuccessful differences. ^d^The number of (X, Y) hits involving the sequence. ^e^The entropy of a position is exponentiated then averaged over each Pfam alignment, distinguishing 6 (not 20) amino acid classes. ^f^Codon degeneracy summed over the protein sequence.

**Table 4:** Mean sequence composition of successful and unsuccessful Pfam protein sets, *F* = −2.

Amino acid	Viral proteins^a^	All proteins			Amino acid 2-mer	Viral proteins^a^	All proteins		
		most successful	least successful	P-value^b^			most successful	least successful	P-value^b^
E	6.6^*c*^	8.2^c^	6.8^c^	0.01	DE	8.3^d^	7.7^d^	4.1^d^	0.00
F	3.4	3.6	4.4	0.02	EE	4.2	7.5	4.1	0.02
H	2.0	1.7	2.1	0.15	EK	2.7	10.1	4.8	0.00
K	6.4	7.0	5.9	0.11	SN	5.4	4.6	1.6	0.00
N	5.5	4.5	3.9	0.17	SE	7.0	7.7	4.5	0.01
Q	4.2	4.2	3.7	0.16	RD	3.5	1.1	3.3	0.02
T	6.2	4.8	5.8	0.05	FA	1.9	1.6	4.0	0.02
W	0.9	0.9	1.5	0.01	FG	1.0	1.3	3.5	0.03
Y	2.5	2.7	3.6	0.01	YR	0.0	0.4	3.1	0.03
G	4.0	5.8	6.8	0.06	YV	0.7	1.1	3.1	0.03

^a^The 20 most successful out of 44. ^b^Significance of the successful/unsuccessful differences. ^c^Amino acid frequencies (%) for the types that differ most between successful/unsuccessful protein sets (10 smallest P-values). ^d^2-mer frequencies (‰) for those that differ most between successful/unsuccessful protein sets (10 smallest P-values).

Differences in codon usage are more striking, especially when comparing the most successful viral proteins (*F* = −2 scheme) to the least successful Pfam proteins (Fig. 7). Four codons are enriched by over 0.01 in the viral proteins (ACT = T, GCT = A, AAC = N, ATT = I) and four depleted (ACG = T, GCG = A, GGC = G, GCC = A). In contrast, in the *F* = 0 scheme (which gave fewer successful designs), the successful/unsuccessful differences are much smaller, with only six codon frequencies shifted by just over 0.005. In the overall set of *F* = −2 successes (Fig. 7, right), many of the viral trends are confirmed, even though this set contains just 13 viral proteins out of 50. Thus, there are codon choices that are more favorable for dual genes, and the corresponding trends are especially visible in the most successful viral proteins.

Finally, given the high success rate for dual gene design and the generality of our algorithm, we used it (with slight adjustments) to design 200 *triple* genes, with three proteins X, Y, Z coded in a fully-overlapping manner. Taking X as the reference, we chose to code Y on the antisense strand in the *F* = −2 frame and Z on the sense strand in the *F* = 1 frame. We tested 20,000 triplets where the corresponding pairs gave high-scoring dual genes. This led to a high success rate: more than 60% of the triplets gave −log *E*_*p*_ > 10. We obtained 200 triplets where all three E-values (X′, Y′, Z’ vs. X, Y, Z, respectively) were better than 10^−20^. Of these, 62 were triplets of non-viral proteins, 30 were triplets of viral proteins, and 40 involved one viral and two non-viral proteins. The DNA and protein sequences for one triplet are shown in Fig. 5. X is bovine, Y is viral, and Z is a bovine tick protein. The E-values vs. Swissprot for the predicted homologs X′, Y′, and Z’ were, respectively, 10^−24^, 10^−23^, and 10^−23^.

## Concluding Discussion

We have shown that for a significant fraction of protein domain pairs, at least 10%, homologs with fully-overlapping coding sequences can be produced rather easily. This contrasts sharply with naive expectations based on the stringent overlap constraint. The fraction of successes obtained is only a lower bound, since we only considered one representative from each Pfam family and only two positions for the X′ 5′-terminus relative to Y′. We also observed that while our algorithm provably maximizes the similarity score *S*(*X*′, *Y*′), a heuristic search that does not reach the maximum can produce additional hits (in other words, maximizing *S*(*X*′, *Y*′) does not always minimize *E*_*p*_(*X*′, *Y*′)). However, our focus here was not to be exhaustive but to demonstrate that overlapping genes could be produced for close homologs of thousands of domain pairs. Since the calculation was done for 125,250 unique pairs, we expect the results are general.

To identify properties that favor overlapping genes, we analyzed the most successful and the least successful of our Pfam proteins. The most striking differences were in codon usage, with 20 codons out of 61 significantly enriched or depleted within the most successful viral proteins, compared to the least successful proteins (with the *F* = −2 overlap scheme; Fig. 7). This may be the result of ancestral selective pressure for gene overlap, even though the modern viral proteins in our dataset do not overlap with each other. Similar trends were found in our larger set of successful proteins, with both the *F* = −2 and *F* = 1 overlap schemes.

The *F* = −2 coding properties can be considered in the light of various hypotheses regarding simpler, ancestral genetic codes that would have contained fewer amino acid types or classes^26–28^. If we express the sequences of the proteins X and Y in the simplified alphabet {Φ, *φ*, Π, *π*}, the simplified sequences can be exactly expressed by a dual gene in the *F* = −2 frame, with the exception of the (*π*, *π*) pairs, which would sometimes be replaced by (*π*, Π) pairs. Thus, in an ancestral world with a simpler {Φ, *ϕ*, Π, *π*} amino acid alphabet, arbitrary overlapping genes could be encoded almost perfectly thanks to the properties of the genetic code^26^, exploited by the *F* = −2 reading frame. This would presumably have been favorable for genome compaction and consequently for viruses, which evolve essentially at fixed capsid volume.

The ease of overlap coding also has implications for mechanisms of gene creation by “overprinting”, in viruses or otherwise^9,29^. The success rate for designing overlapping coding schemes for the 44 viral proteins considered here was remarkably high: almost 45% of the considered pairs gave designs with E-values better than 10^−15^ vs. the natural proteins. It could be of interest to test whether the modern genetic code is especially tolerant of overlapping genes, compared to ancestral codes (including competing codes that did not survive), or artificial codes (including codes that use larger or smaller amino acid alphabets or codons of a different length). This could be tested by repeating our calculations using one or more alternative codes and comparing the level of success obtained to the values reported above. Such comparisons could help determine whether a high tolerance for overlapping coding schemes played a role in the selection of the genetic code.

Another hypothesis related to gene creation is the Rodin-Ohno hypothesis for the appearance of the two aminoacyl-tRNA synthetase classes, proposed to have occurred on the sense:antisence strands of an overlapping gene^30,31^. Statistical evidence for such an ancestral dual gene was found by analyzing the DNA sequences of modern aminoacyl-tRNA synthetases^32^. A hypothetical dual gene that exhibits a weak similarity to both synthetase classes was discovered in the mold *Achlya klebsiana* and several other organisms^33,34^. The recent design of two overlapping synthetase genes^13–16^ also provides some support for this hypothesis.

Our method should allow overlapping genes to be designed and produced readily for applications. This could be of technological interest, for example to produce a very compact plasmid or to prevent the fixation of mutations and genetic drift in a plasmid or a synthetic organism. Given the surprising ease with which a dual gene can be designed, we also considered and verified the possibility of creating 200 triple genes that use not two, but three of the six possible overlapping reading frames. With additional effort, other triple genes with even better E-values could be obtained. This could be a way to reduce genetic drift even more drastically, constraining an artifical organism to stay close to its original genotype.

## Electronic supplementary material

Supplementary Information

## References

[1] Rogozin IB (2002). Purifying and directional selection in overlapping prokaryotic genes. Trends Genet..

[2] Kumar A (2009). An overview of nested genes in eukaryotic genomes. Euk. Cell.

[3] Behura SK, Severson DW (2013). Overlapping genes of *Aedes aegypti*: evolutionary implications from comparison with orthologs of *Anopheles gambiae* and other insects. BMC Evol. Biol..

[4] Saha D, Panda A, Podder S, Ghosh TC (2015). Overlapping genes: a new strategy of thermophilic stress tolerance in prokaryotes. Extremophiles.

[5] Cassan E, Arigon-Chiffoleau AM, Mesnard JM, Gross A, Gascuel O (2016). Concomitant emergence of the antisense protein gene of HIV-1 and of the pandemic. Proc. Natl. Acad. Sci. USA.

[6] Faure E, Tribolo S, Levasseur A, Seligmann H, Barthelemy RM (2011). Probable presence of an ubiquitous cryptic mitochondrial gene on the antisense strand of the cytochrome oxidase I gene. Biol. Direct.

[7] Rancurel C, Khosravi M, Dunker AK, Romero PR, Karlin DG (2009). Overlapping genes produce proteins with unusual sequence properties and offer insights into de novo protein creation. J. Virol..

[8] Sabbath N, Wagner A, Karlin DG (2012). Evolution of viral proteins originated *de novo* by overprinting. Mol. Biol. Evol..

[9] Pavesi A, Magiorkinis G, Karlin DG (2013). Viral proteins originated *de novo* by overprinting can be identified by codon usage: application to the “gene nursery” of deltaretroviruses. PLoS Comp. Bio..

[10] Grass’e, P. P. *Evolution of living organisms*: *evidence for a new theory of transformation* (Academic Press, New York, 1977).

[11] Zull JE, Smith SK (1990). Is genetic code redundancy related to retention of structural information in both dna strands?. Trends Biochem. Sci..

[12] Goldstein A, Brutlag DL (1989). Is there a relationship between DNA sequences encoding peptide ligands and their receptors?. Proc. Natl. Acad. Sci. USA.

[13] Pham Y (2007). A minimal TrpRS catalytic domain supports sense/antisense ancestry of class I and II aminoacyl-tRNA synthetases. Molec. Cell.

[14] Li L, Weinreb V, Francklyn C, Carter CW (2011). Histidyl–tRNA urzymes class I and II aminoacylt-tRNA urzymes have comparable catalytic activities for cognate amino acid activation. J. Biol. Chem..

[15] Li L, Francklyn C, Carter CW (2013). Aminoacylating urzymes challenge the RNA world hypothesis. J. Biol. Chem..

[16] Martinez-Rodriguez L (2015). Functional class I and II amino acid-activating enzymes can be coded by opposite strands of the same gene. J. Biol. Chem..

[17] Lèbre S, Gascuel O (2017). The combinatorics of overlapping genes. J. Theor. Biol..

[18] Finn RD (2008). The Pfam protein families database. Nucl. Acids Res..

[19] Durbin, R., Eddy, S. R., Krogh, A. & Mitchison, G. *Biological sequence analysis* (Cambridge University Press, Cambridge, United Kingdom, 2002).

[20] Boursnell M, Binns MM, Brown TDK (1985). Sequencing of coronavirus IBV genomic RNA: Three open reading frames in the 5′ “unique” region of mRNA D. J. Gen. Virol..

[21] Pelet T, Curran J, Kolakofsky D (1991). The P gene of bovine parainfluenza virus 3 expresses all three reading frames from a single mRNA editing site. EMBO J..

[22] Root-Bernstein R, Root-Bernstein M (2016). The ribosome as a missing link in prebiotic evolution II: ribosomes encode ribosomal proteins that bind to common regions of their own mRNAs and rRNAs. J. Theor. Biol..

[23] Seligmann H (2017). Natural mitochondrial proteolysis confirms transcription systematically exchanging/deleting nucleotides, peptides coded by expanded codons. J. Theor. Biol..

[24] Wilson D, Madera M, Vogel C, Chothia C, Gough J (2007). The SUPERFAMILY database in 2007: families and functions. Nucl. Acids Res..

[25] Andreeva A (2004). SCOP database in 2004: refinements integrate structure and sequence family data. Nucl. Acids Res..

[26] Delarue M (2007). An asymmetric underlying rule in the assignment of codons: possible clue to a quick early evolution of the genetic code via successive binary choices. RNA.

[27] Lehmann J, Cibils M, Libchaber A (2015). Emergence of a code in the polymerization of amino acids along RNA templates. PLoS One.

[28] Carter CW, Wolfenden R (2015). tRNA acceptor stem and anticodon bases form independent codes related to protein folding. Proc. Natl. Acad. Sci. USA.

[29] Delaye L, DeLuna A, Lazcano A, Becerra A (2008). The origin of a novel gene through overprinting in Escherichia coli. BMC Evol. Biol..

[30] Rodin SN, Ohno S (1995). Two types of aminoacyl-tRNA synthetase could be originally encoded by complementary strands of the same nucleic acid. Orig. Life Evol. Biosph..

[31] Carter CW (2014). The Rodin-Ohno hypothesis that two enzyme superfamilies descended from one ancestral gene: an unlikely scenario for the origins of translation that will not be dismissed. Biol. Direct.

[32] Chandrasekaran SN, Yardimci GG, Erdogan O, Roach J, Carter CW (2013). Statistical evaluation of the Rodin-Ohno hypothesis: Sense/antisense coding of ancestral class I and II aminoacyl-tRNA synthetases. Molec. Biol. Evol..

[33] Carter CW, Duax W (2002). Did tRNA synthetase classes arise on opposite strands of the same gene?. Molec. Cell.

[34] Williams TA, Wolfe KH, Fares MA (2009). No Rosetta stone for a sense/antisense origin of aminoacyl-tRNA synthetase classes. Molec. Biol. Evol..

